# Evaluating the efficacy of digital media platforms in disseminating public health information: A global review with implications for South Africa

**DOI:** 10.4102/phcfm.v18i1.5182

**Published:** 2026-02-04

**Authors:** Hulisani Matakanye, Ndivhuwo D. Sundani

**Affiliations:** 1 Department of Health Studies, Faculty of Human Science, University of South Africa, Pretoria, South Africa; 2 Department of Communication Science, Faculty of Human Science, University of South Africa, Pretoria, South Africa

**Keywords:** digital media, health information dissemination, health literacy, health promotion, public health information, social media, South Africa

## Abstract

**Background:**

This study employs a non-empirical review to examine how digital media platforms support public health education and awareness, with specific relevance to the South African context.

**Aim:**

To explore the use of digital media in disseminating public health information and its implications for improving citizens’ awareness and health literacy. Drawing on global evidence, the review highlights relevance for South Africa’s digital communication landscape.

**Method:**

The review followed Preferred Reporting Items for Systematic Reviews and Meta-Analyses guidelines. A comprehensive search was conducted across databases including Google Scholars, EBSCOhost, PubMed, ScienceDirect, Sabinet, ProQuest, Scopus and MEDLINE. Studies were selected using predefined inclusion and exclusion criteria, resulting in 15 peer-reviewed full text articles published between January 2019 and March 2023. Quality appraisal was conducted using the Joanna Briggs Institute Qualitative Assessment and Review Instrument, and findings were synthesised thematically.

**Results:**

Findings indicate that digital media use reduces misinformation, strengthens social support networks, enhances the reach and efficiency of health information, and promotes positive health behaviours. Improvements in health knowledge and engagement were observed across diverse populations. However, challenges such as digital literacy gaps, unequal access to digital platforms, and algorithmic bias remain significant concerns.

**Conclusion:**

Digital media represents a transformative tool for public health communication. When strategically implemented, it enhances awareness, supports behaviour change, and strengthens public engagement with health information.

**Contribution:**

The review highlights the importance of collaboration between health professionals and digital communication specialists to optimise digital strategies. It underscores policy imperatives such as improving digital literacy, ensuring equitable access, strengthening regulation of online health information, and fostering public-private partnership to improve health outcomes in South Africa.

## Introduction and scope of the study

The World Health Organization (WHO) defines health as a state of complete physical, mental, and social well-being, not merely the absence of disease.^1^ The Ottawa Charter for Health Promotion further defines health promotion as the process of enabling individuals to gain control over and improve their health.^2^ This broad approach to health promotion spans multiple disciplines, including medicine, public health, sociology, psychology, and economics, to guide interventions that aim to improve community health.^3^ Health promotion goes beyond disease prevention; it encompasses creating supportive environments, developing public health policies, enhancing personal skills, reorienting health services, and fostering multisectoral community action.^4,5,6^

As the global disease burden continues to rise, health promotion has become a critical strategy in tackling its root causes.^7^ Social media has emerged as a powerful communication tool, with We Are Social projections estimating that by 2020, social media users would represent half of the global population.^8^ Given the widespread use of social media, its potential in public health promotion has become undeniable, especially considering the challenges faced during health crises like the COVID-19 pandemic.^9^

Social media platforms have simplified the search for health information, especially during crises, where individuals increasingly rely on them for timely updates and information.^10,11^ Research has shown that digital health promotion can bridge gaps in healthcare access, reinforce mass media campaigns, and influence societal norms around health risk behaviours.^12^ Platforms like Facebook also facilitate peer connections, as evidenced by cancer patients’ use of social media to find support and information.^13^ However, while digital media holds promise for health promotion, challenges related to digital literacy remain, preventing meaningful behavioural change for some populations.^14^

The media also plays a crucial role in shaping political narratives, with social media contributing to the dissemination of political ideologies that may sometimes marginalise specific groups.^8^ In this context, guiding users to critically evaluate credible health information becomes crucial in combating the spread of misinformation, which can hinder public health efforts.^15^ Social media has also been criticised for facilitating the spread of false information, particularly during the pandemic, which has raised concerns about its impact on public health.^16,17^ Therefore, it is essential to explore both the benefits and drawbacks of social media in the context of public health promotion.

Despite its challenges, social media offers unprecedented opportunities for real-time engagement with diverse populations. Health promotion strategies must evolve to leverage the full potential of digital media, integrating multi-disciplinary approaches that include education, health policy, and digital literacy.^18^ For example, increasing digital literacy among older adults is crucial to improving their ability to assess health news accurately.^19^ Additionally, expanding access to health information technology can enhance efficiency, reduce healthcare costs, and improve global access to healthcare.^20^ By integrating these technologies into healthcare practice, we can maximise their impact on public health promotion.

The intersection of digital media and political factors also plays a critical role in shaping health outcomes. Political dynamics, as seen in campaigns such as those targeting vaccine hesitancy during the COVID-19 pandemic, highlight the importance of understanding trust and misinformation.^21^ Political analyses can reveal patterns of trust and mistrust, which can guide more effective health communication strategies, particularly in the context of vaccine campaigns in regions like Sierra Leone.^22^ By supporting policies that promote digital inclusivity and infrastructure development, governments can improve access to health information and foster transparency in health messaging.^23^

Digital media platforms offer expanding opportunities for public health promotion. Although global research forms the primary evidence base, this review interprets these insights through a South African lens, where digital media uptake is growing but remains uneven. By drawing on international patterns related to internet access, multilingual communication, engagement dynamics, and digital literacy disparities, the review highlights implications for South Africa’s distinct socio-technical environment. The country presents a particularly relevant case because of its progressive yet unevenly implemented policies, such as the National Digital Health Strategy and the National Health Insurance framework, alongside persistent challenges including urban-rural connectivity gaps, limited digital literacy, and a dual burden of communicable and non-communicable diseases. Although social media use is widespread, it remains underutilised in formal health promotion, creating a mismatch between digital potential and policy execution. Addressing these gaps is essential for strengthening national and regional digital health communication strategies across the Global South.

## Research methods and design

This review synthesised global peer-reviewed literature on digital media use in public health communication, highlighting patterns across diverse regions. International evidence was used to identify implications for South Africa, where digital media access and adoption remain uneven. This study was guided by the Preferred Reporting Items for Systematic reviews and Meta-Analyses (PRISMA) framework to ensure transparency and methodological rigour^24^ as detailed by Matakanye and Mboweni^25^ While the review adhered to the PRISMA principle for literature identification, screening and reporting, the synthesis adopted a qualitative and descriptive approach using thematic analysis rather than a meta-analytical or statistical aggregation of results.^24,26^

### Search strategy

A comprehensive literature search was conducted across major global databases, including EBSCOhost, PubMed, ScienceDirect, ProQuest, Cambridge Core, Scopus, Nursing/Academic Edition, Humanities and Social Sciences Index Retrospective Humanities Sources, MasterFILE Premier, MEDLINE, and SociINDEX, to identify peer-reviewed studies on the use of digital media for public health promotion and information dissemination. To ensure representation of African scholarship, region-specific databases such as Sabinet and Africa-Wide Information were included. For conceptual clarity, the review defines digital media as all internet-based communication technology, social media as interactive user-generated content platforms (Facebook, X [formerly Twitter] and Instagram), and online platforms as broad digital tools such as websites and applications; these definitions are applied consistently throughout the article.

**FIGURE 1:**
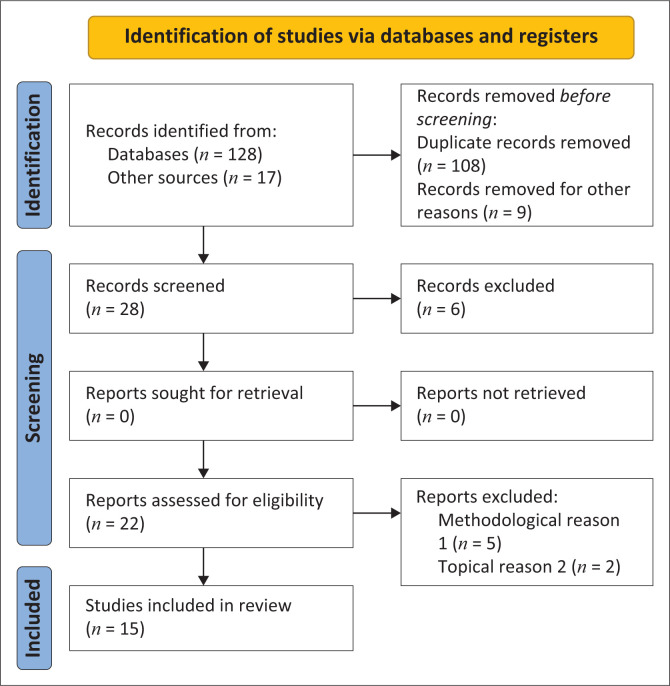
Systematic review flow chart adapted from preferred reporting items for systematic review – Preferred Reporting Items for Systematic Reviews and Meta-Analyses.

The search strategy was designed to examine how digital media support public health information dissemination and community awareness. The review focused on studies published between January 2019 and March 2023 to capture the most recent evidence shaped by the rapid digital transformation intensified during the COVID-19 pandemic. This period reflects major shifts in This period reflects a major shifts in communication practices across the Global South, including South Africa, due to the expansion of digital media, making earlier studies less relevant to the contemporary digital landscape. Only peer-reviewed English language articles were included to ensure methodological quality and accessibility. The detailed search strategy is illustrated in Figure 1.

The search used Boolean combinations of controlled vocabulary and free-text terms including:

(“digital media” OR “social media” OR “online platforms” OR “Facebook” OR “Twitter” OR “YouTube” OR “Instagram” OR “TikTok”)

AND (“public health communication” OR “health promotion” OR “health education” OR “health information dissemination”)

AND (“awareness” OR “behaviour change” OR “engagement” OR “health outcomes”)

### Screening and study selection

The screening process occurred in three phases: identification, eligibility assessment and inclusion as illustrated in Figure 1. The initial database search retrieved 145 records. After removing duplicates (*n* = 37), 108 unique articles remained. Titles and abstracts were screened for relevance, resulting in the exclusion of 80 articles that did not meet the inclusion criteria. Twenty-eight articles were then assessed for eligibility based on methodological quality and relevance to the review question. Following full-text review, 13 articles were excluded because of insufficient methodological detail or outcomes outside the study scope. The final review included 15 articles that met all inclusion criteria.

To ensure consistency, the inclusion criteria required studies that:

- Examined the use of digital or social media platforms for public health promotion or public health information dissemination.
- Reported measurable or thematic outcomes related to behaviour change, awareness or health literacy.
- Employed a qualitative, quantitative or mixed-method design.
- Were published from January 2019 to March 2023.
- Were written in English focusing on digital media and public health information dissemination.

Exclusion criteria included:

- Studies published before 2019 or after March 2023.
- Articles lacking a defined methodology or peer-review.
- Non-English publication.

### Quality appraisal

All 15 studies were appraised using the Joanna Briggs Institute’s (JBI’s) Qualitative Assessment and Review Instrument (QARI) tool, which evaluates 10 methodological domains and assigns scores up to 10, with studies classified as high (8–10), moderate (6–7) or low quality (≤ 5). Only moderate and high-quality studies were included in the synthesis, except for low-scoring studies that provided essential contextual insights relevant to South Africa. Two independent reviewers conducted the appraisal, compared scores, and achieved substantial inter-rated agreement (Cohen’s kappa = 0.86), resolving any discrepancy through discussion with a third reviewer. The final QARI ratings are presented in Appendix 1 – Table 1-A1.

### Data extraction and synthesis

From the final selection of 15 studies, relevant information was extracted into a structured Excel matrix capturing author, year, country, study design, population, objectives, and key findings. Data were synthesised descriptively to summarise patterns across study characteristics and outcomes related to the use of digital media for public health promotion. To ensure methodological rigour, the JBI Critical Appraisal Checklist was used to assess study quality. Only studies meeting acceptable quality thresholds (score ≥ 5) were retained for synthesis. Two reviewers independently conducted extraction and appraisal, with discrepancies resolved through consensus. The researchers focused on organising, comparing, and summarising the extracted data to provide a comprehensive overview of the scope, quality, and context of existing research before deeper interpretive analysis.

### Thematic data analysis

Following data synthesis, a thematic analysis was conducted to identify and interpret recurring concepts, relationships, and insights across the studies. The qualitative synthesis was guided by Braun and Clarke’s framework, allowing integration of findings from both qualitative and quantitative studies.^27,28^

The process involved six key steps:

- Familiarisation: The researchers reviewed all extracted data and study summaries to identify preliminary ideas and patterns.
- Coding: A coding framework was developed, combining inductive codes emerging from the data and deductive codes aligned with the research question.
- Theme development: Related codes were grouped into broader categories representing patterns in how digital media influences public health awareness and engagement.
- Review and refinement: Themes were reviewed and revised for internal consistency, distinctness, and alignment with the data.
- Validation: An independent reviewer verified the coherence and completeness of the final themes to ensure analytic rigour.
- Interpretation and reporting: The finalised themes were interpreted in relation to existing literature and research questions, highlighting implications, consistencies and gaps.

### Ethical considerations

Ethical approval was not required because the review used publicly available data and involved no human participants. However, ethical principles were maintained through transparent methods, adherence to inclusion criteria, proper citation practices, and detailed documentation to support reproducibility. The review also followed PRISMA guidelines to ensure accuracy, clarity and ethical reporting. This article followed all ethical standards for research without direct contact with human or animal subjects.

## Results

### Characteristics of reviewed sources

The PRISMA flowchart (Figure 1) summarises the selection process. A total of 145 records were identified, from which duplicates and irrelevant sources (e.g. abstract, non-academic publication) were removed, leaving 28 studies for eligibility screening. After full-text review, 15 studies met the inclusion criteria, while 7 were excluded for methodological or topical reasons.

The included studies were published between 2019 and 2023, with most appearing in 2020–2022. Research designs included qualitative (40%), systematic reviews (20%), and quantitative or mixed-method studies (approximately 13%), along with a few observational and case study designs.

Geographically, studies originated from diverse settings: the United States (40%), South Africa (13.3%), Australia (13.3%) and smaller contributions from India, Iran, Italy, the United Kingdom, and Singapore, each representing 6.6% of the total. Most examined how social or digital media platforms supported health communication during the COVID-19 pandemic, with a focus on topics such as tuberculosis (TB), mental health, and general public health awareness. Detailed summaries of individual studies are presented in Appendix 1 – Table 1-A1.

### Thematic results

Analysis identified five overarching themes describing how digital media platforms are used to enhance public health promotion: (1) reducing public health misinformation; (2) enhancing social support; (3) providing health information to the community; (4) facilitating and promoting healthy behaviours; and (5) improving health-related outcomes. These themes are summarised in Table 1.

**TABLE 1:** Themes and sub-themes for the efficacy of digital media platforms in disseminating public health information.

Themes	Sub-themes
1. Reducing the risk of public health misinformation	1.1 Amplify awareness 1.2 Bridge the knowledge gap
2. Enhance social support	2.1 Providing emotional and informational support 2.2 Reduce stigma and promote inclusion
3. Provision of health information	3.1 Expanding access and reach 3.2 Targeting specific populations 3.3 Influencer-based communication
4. Facilitating and promoting healthy behaviour	4.1 Encouraging preventative and healthy practice 4.2 Supporting behavioural change through education
5. Improving health-related outcomes	5.1 Enhancing treatment compliance 5.2 Enhancing help-seeking and self-care

#### Theme 1: Reduce the risk of public health misinformation

**Sub-theme 1.1 Amplifying awareness:** Multiple studies demonstrated that social media campaigns increase public awareness and reach large audiences. Studies reported substantial improvements in engagement metrics, including increased post reach and user interaction, following influencer-led and organisational digital media campaigns.^29,30^ One study observed similar outcomes in South Africa, where online platforms provided cost-effective dissemination of accurate health information.^18^

**Sub-theme 1.2 Bridge the knowledge gap:** Findings from this study show that disseminating high-quality public health information on various social media platforms can greatly benefit users by providing access to specific advice and solutions on health-related topics. This practice helps bridge knowledge gaps and improves community functional health literacy. Studies^31,32^ found that inconsistent or unclear online messages limited comprehension among young audiences. Conversely, structured campaigns supported by health professionals improved message clarity and bridged knowledge gaps across the population.

#### Theme 2: Enhance social support

**Sub-theme 2.1 Providing emotional and informational support:** Study results found that social media platforms enable individuals to exchange emotional and informational support. One study found that online peer groups among young adult cancer survivors enhanced motivation and engagement in health activities.^33^ Similarly, another study reported that older adults used social media to maintain social connectedness and access reliable health information.^19^

**Sub-theme 2.2 Reduce stigma and promote inclusion:** Study findings show that several studies highlighted the role of digital platforms in reducing stigma associated with specific conditions. Studies have noted that mental health and maternal health campaigns delivered through social media reached a wide audience and normalised health conversations, supporting more inclusive participation.^12,34^

#### Theme 3: Provision of health information

**Sub-theme 3.1 Expanding access and reach:** Studies consistently reported that social media extends access to health information. One study identified diverse communication strategies among non-profit organisations that increased message reach.^35^ Another study found that Facebook pages dedicated to tuberculosis awareness served as accessible sources of health promotion content.^18^

**Sub-theme 3.2 Targeting specific populations:** Campaigns using tailored messaging reached key demographic groups effectively. One study has achieved substantial engagement from youth audiences,^34^ while another study reported increased awareness of influenza vaccination among specific regional populations.^29^

**Sub-theme 3.3 Influencer-based communication:** This study highlighted that the use of influencers enhances visibility and engagement. Studies showed that collaboration with online figures improved content dissemination and message retention among young audiences.^30,32^

#### Theme 4: Facilitating and promoting healthy behaviour

**Sub-theme 4.1: Encouraging preventive and healthy practices:** This study found that digital interventions were effective in motivating users to adopt preventive behaviours. One study reported increased physical activity and improved knowledge among women participating in digital health programmes.^36^ Another study found that online promotion through dating apps led to higher HIV self-testing uptake.^37^

**Sub-theme 4.2 Supporting behavioural change through education:** This study found that educational campaigns using digital media enhance awareness and positive attitudes towards healthy behaviours, especially among younger and more digitally active populations.^32,38^

#### Theme 5: Improving health-related outcomes

**Sub-theme 5.1 Enhancing treatment compliance:** This study discovered that digital media enhances treatment adherence. Studies included in this review show that visual and interactive social media campaigns supported treatment adherence, particularly in tuberculosis care initiatives.^18,39^

**Sub-theme 5.2 Enhancing help-seeking and self-care:** The findings of this study show that digital media promotes help-seeking and self-care. One study observed that older adults that use social media improved engagement in self-care and wellbeing activities.^19^ Other studies found that online interventions facilitated participation in health-related activities such as physical exercise, and led to enhanced perinatal care and breastfeeding promotion.^12,33^

## Discussion

The review examined how digital media platforms are used to disseminate public health information and promote healthy behaviours, with a particular focus on South Africa. Consistent with findings from the study conducted by Ghahramani, de Courten, and Prokofieva,^38^ the findings confirm that social media platforms can enhance the reach, visibility, and timeliness of health promotion messages. They enable rapid communication during health crises, increase engagement with younger and digitally active populations, and provide opportunities for interactive education that traditional media often lack. Although this review emphasises South Africa, the available evidence is largely global, with only a small number of South African studies; therefore, interpretations of country-specific implications are informed by broader international patterns unless otherwise stated.

However, while these advantages are well-documented, the evidence also reveals significant limitations and contradictions in digital health promotion outcomes. For instance, a study by Acha-Anyi et al.^18^ demonstrated the success of Facebook-based campaigns such as TB proof in South Africa in raising awareness and promoting treatment adherence; yet the same study cautions that reach and impact are unevenly distributed. Populations with low digital literacy, limited internet access, or language barriers remain excluded from interventions, reflecting the persistent digital divide that affects health equity in many low- and middle-income settings.

Similarly, while influencer-led campaigns, such as those described by Bozzola et al.^30^ and Bonnevie et al.^29^ achieved substantial engagement and positive attitudinal shifts towards vaccination, these results depend heavily on algorithmic amplification and content visibility determined by platform design rather than public health priorities. This raises concerns about algorithmic bias, which may inadvertently prioritise entertainment-oriented or sensational content over credible health information.

The literature also highlights that exposure does not guarantee comprehension or behaviour change. As Peyman et al.^36^ and Ghahramani et al.^38^ note, while digital interventions improve short-term awareness and attitudes, sustained behavioural change requires deeper structural support, health system integration, and reinforcement through offline community engagement. In South Africa, where health literacy levels and access to digital infrastructure vary widely, these factors present tangible barriers to achieving equitable outcomes from digital health promotion.

Furthermore, the risk of misinformation remains a critical concern. Although health professionals can use social media to counter false narratives, as noted by Acha-Anyi et al.,^18^ and Harris et al.,^32^ the sheer volume and velocity of information online make it difficult to control misleading or harmful content. This challenge is exacerbated during crises, such as the COVID-19 pandemic, when uncertainty and fear fuel the viral spread of misinformation.

Despite these challenges, several studies^19,35^ emphasise the value of social media in fostering social support networks that complement clinical interventions. Peer-to-peer interactions, patient advocacy groups, and virtual communities provide emotional and informational support that can enhance mental health outcomes and adherence to treatment. One study found that integrating wearable technologies and social platforms improved motivation and engagement among cancer survivors.^33^

Health professionals also benefit from digital platforms as low-cost, high-reach community channels. Studies affirm that social media enables healthcare workers to disseminate accurate information efficiently, engage with patients, advocate for healthy behaviours, and promote public awareness campaigns.^35,40^ Evidence from one study^36^ further demonstrates that multimedia and text-based interventions can enhance knowledge and physical activity among women, reinforcing the potential of digital media to support behaviour change.

From a policy perspective, the findings indicate that digital health strategies must extend beyond platform use to include capacity-building initiatives that enhance digital literacy, promote ethical content moderation, and ensure equitable access to digital infrastructure. In South Africa, this means aligning social media-based health promotion with broader public health and information and communication technology (ICT) policies to address systemic inequalities that influence digital participation.

Utilising these platforms enables health professionals to rapidly disseminate information, reach target populations, and counteract prevalent health-related misinformation circulating on social media. Furthermore, digital media campaigns encourage health-promoting behaviours and empower individuals with valuable health information, thereby fostering social support and enhancing individual health outcomes within communities.^41^

### Implications of the study

The study’s findings underscore the importance of policymakers to integrate public health campaigns into social media platforms. Leveraging social media can ensure the widespread dissemination of accurate health information at a larger scale. By integrating campaigns into social media, policymakers can extend the reach and accessibility of crucial health messages, engage a broader audience and promote informed decision-making regarding health behaviours.

Moreover, the study adds to the existing body of knowledge by highlighting the benefits of the Department of Health (DoH) utilising social media as a potent tool for public health awareness campaigns. Policymakers can utilise this evidence to refine communication strategies, prioritising digital media platforms as key components of health promotion initiatives.

### Limitations of the study and future research direction

The study focused solely on full-text, peer-reviewed research published in peer-reviewed journals from January 2019 to March 2023. Studies predating 2019 and those not meeting the peer-reviewed criteria were excluded. Expanding the scope to include additional studies might have yielded different outcomes for the review.

### Future directions for dealing with digital media platforms in disseminating public health information in South Africa

The National Department of Health ought to reassess its health promotion strategies and establish guidelines and protocols incorporating digital media as a pivotal tool for heightening public health awareness and extending outreach to a broader demographic at reduced expenses. It is crucial that social media health promotion campaigns are meticulously crafted to align with the various stages of behaviour change, ensuring they offer actionable information tailored to the needs of communities.

## Conclusion and recommendation

Digital media plays a significant yet complex role in health promotion, offering a powerful avenue for disseminating information, fostering social support, and encouraging healthier behaviours. However, its effectiveness is shaped by broader political and social dynamics, particularly the spread of misinformation and varying levels of public trust in health sources. To maximise the benefits of digital media, health strategies must prioritise digital literacy, inclusion of marginalised groups, and strong mechanisms to counter misinformation. Supportive government policies that enhance transparency and digital infrastructure are essential. By acknowledging both the strengths and limitations of digital platforms, public health campaigns can better meet the diverse needs of communities and promote sustained engagement in health-related behaviours.

Based on these findings, the study recommends strengthening political and policy support through targeted investments in digital infrastructure, enhancing digital literacy, and promoting inclusive access to online health information. Enhanced collaboration between health professionals, digital media experts, and policymakers is essential for designing evidence-based context-appropriate public health campaigns. Incorporating political analysis into health communication strategies is also crucial as understanding political dynamics can help address misinformation, build trust, and tailor interventions to diverse communities. Future research should evaluate the long-term effectiveness of digital media-based health promotion, ensuring that such initiatives translate into sustained behaviour change rather than short-term awareness gains.

## Appendix 1

The current study included eleven scholarly documents retrieved from various databases. Table 1-A1 illustrates the researcher’s complete search process.

**TABLE 1-A1:** Summary of the scholarly documents used in the study extracted and synthesised from original studies with quality assessment scores, adapted from the Qualitative Assessment and Review Instrument tool.

Author and Year	Country	Purpose	Participants	Methodology (Data collection and analysis)	Key findings	QARI score
Acha-Anyi et al.^18^	South Africa	Social media for health promotion: A visual analysis of ‘TB proof’ South Africa’s Facebook page.	Observation on TB Proof South Africa’s Facebook page.	A qualitative case study approach was used. An in-depth visual analysis of tuberculosis (TB) Proof South Africa’s Facebook page was carried out over a 5-month period.	Themes were identified, TB medication, TB patients, and healthcare workers raising awareness of TB. The findings have potential implications for health promotion efforts using social media.	8
Latha et al.^34^	India	Effective use of social media platforms for the promotion of mental health awareness.	Social media viewers who were willing to participate.	Qualitative study and descriptive statistics, such as frequency and proportions, were computed for the number of likes and shares.	The Facebook and Instagram posts concerning all the campaigns brought about a considerable amount of reach to the targeted population. After the campaigns, the page reached around 10.3k people.	6
Peyman et al.^36^	Iran	Digital Media-based Health Intervention for the Promotion of Women’s physical activity: a quasi-experimental study.	360 women were divided into case and control groups.	Quasi-experimental study. Data were collected using a questionnaire and analysed using SPSS 20.	The mean scores of knowledge, attitude, and level of physical activity in the control group were not significantly different before and after the intervention. In the case group, there was a significant difference as the mean score increased.	7
Bailey et al.^41^	Australia	The mental health and social media use of young Australians during the COVID-19 pandemic.	371 young people aged 16–25 years.	A quantitative cross-sectional anonymous community survey was advertised via social media, and the data were analysed using frequencies and percentages.	Participants reported high levels of psychological distress, with over 40% reporting severe levels of anxiety and depression. Social media use was high, with 96% reporting use at least once a day, and two-thirds reporting an increase in social media use since the start of the pandemic.	7
Miropolsky et al.^42^	United States	Participant perceptions of a Fitbit and Facebook intervention for young adult cancer survivors: A qualitative study.	13 young adult cancer survivors aged 20–39 years.	Qualitative approach. Data were collected using semi-structured qualitative interviews, and thematic analysis was used to analyse the data.	Participants described the wide-ranging benefits of the intervention, citing the Fitbit device and buddy system as major motivators toengage in physical activity (PA) and reach goals.	6
Vedel et al.^35^	Canada, United States	Social Media strategies for health promotion by non-profit organisations: Multiple case study design.	American non-profit cancer organisations engaged in the use of social media for health promotion.	The study conducted a multiple case study of American non-profit cancer organisations using social media for health promotion. Data were collected using a qualitative approach, including in-depth interviews and content analysis of each social media strategy. The study employed thematic and comparative analysis to examine how social media was used to achieve health promotion goals.	The resulting process model demonstrates how social media strategies are enacted by non-profit organisations to achieve health promotion goals. They put forth three types of social media strategies relative to their use of existing information and communication technologies, namely, replicate, transform, or innovate, each affecting the content, format, and delivery of the message differently.	6
Durowaye et al.^12^	Canada, United States	Public health perinatal promotion during the COVID-19 pandemic: a social media analysis.	Canadian provincial, municipal, and non-governmental organisation (NGO) websites, with emphasis on jurisdictional and geographical breadth and the availability of organisational websites.	Lesson learned: Perinatal health promotion. The content of websites and Facebook posts from a multijurisdictional and geographically diverse sample of government and NGO was evaluated using thematic content analysis in 2020.	Major Facebook perinatal health promotion themes included breastfeeding, infant care, labour and delivery, parenting support, and a healthy pregnancy. Facebook’s COVID-19-themed perinatal health promotion peaked in the second quarter of 2020. Websites emphasised COVID-19 transmission routes, disease severity, and the need for infection control during pregnancy and infant care, whereas Facebook posts focused on changes to local health services, including visitor restrictions. NGO perinatal health promotion refers to organisations’ individual mandates.	6
Stafylis et al.^37^	United States	Relative Effectiveness of social media, dating apps, and information search sites in promoting HIV self-testing: Observational cohort study.	254 MSM aged 18-30 years who identified as Latinx or black or African American people.	Observational cohort study	177 (69.7%) ordered a home HIV self-test kit. Most of the self-test kits were ordered by participants enrolled in dating apps. Data show that using popular dating apps might be an efficient way to promote HIV self-testing.	7
Ghahramani et al.^38^	Australia	The potential of social media in health promotion beyond creating awareness: an integrative review.	Original papers published between January 2010 and April 2022	Systematic review	Overall, the results show that though social media has potential for promoting behaviour change, the estimation of this change in the long term lies outside the scope of social media health campaigns.	7
Kubheka et al.^40^	South Africa	Social media health promotion in South Africa: Opportunities and challenges.	Articles on health promotion using social media.	Systematic review	Social media has the potential to be an effective health promotion tool in South Africa. It presents an opportunity for scaling health promotion programmes because of its low-cost, its ability to have virtual communities, and the ease of access, eliminating geographical barriers.	7
Bozzola et al.^30^	Italy	Social media use to improve communication on children’s and adolescents’ health: the role of the Italian Paediatric Society influencers.	20 groups of paediatric Influencers (11 females, 9 males) aged 29–48 years.	Lesson learned: PI were asked to share Facebook messages from the official Facebook page of SIP, including the link to the specific campaign or post, and were evaluated	A significant increase in Facebook daily new likes, page engagement, and total reach, as well as in lifetime post total and organic reach, was evidenced. As for PI, they reported a positive experience in most cases.	7
Cheng et al.^31^	United States	The Role of Tailored Public Health Messaging to Young Adults During COVID-19: ‘There’s a lot of ambiguity around what it means to be safe’.	50 young adults residing in British Columbia, Canada, were recruited to participate in a focus group in groups of four to six.	Qualitative study: Focus group discussions were used to collect data, and thematic analysis was used for analysis.	Findings show that young adults face confusion due to inconsistent messaging and are not reached due to the ineffectiveness of existing strategies.	6
Bonnevie et al.^29^	United States	Using social media influencers to increase knowledge and positive attitudes towards the flu vaccine.	117 influencers were recruited.	Quantitative cross-sectional pre- and post-campaign surveys	Results from the region that received the campaign showed significant increases in positive beliefs about the flu vaccine and significant decreases in negative community attitudes towards the vaccine.	7
Harris et al.^32^	United Kingdom	Young people’s experiences and perceptions of YouTuber-produced health content: implications for health promotion.	85 young people (13–18 years old) were recruited from schools.	Qualitative approach: Focus groups (November 2017 to January 2018)	The study confirms that YouTuber health content was one of the many sources of health information used by young people and was most frequently encountered during their routine viewing. Collaboration between public health organisations and YouTubers could be promising for communicating health messages to young people.	8
Han et al.^19^	Singapore	Impact of social media on health-related outcomes among older adults in Singapore: A qualitative study.	Older adults (*N* = 16) who were aged between 60 and 80 years.	Qualitative research design. In-depth interviews were conducted. Employing a thematic analysis	Two themes emerged: personal attitude and social influence. This study’s findings provide practical insights into how social media can be deployed to improve health-related outcomes in older adults.	7

Note: Please see the full reference list of the article Matakanye H, Sundani ND. Evaluating the efficacy of digital media platforms in disseminating public health information: A global review with implications for South Africa. Afr J Prm Health Care Fam Med. 2026;18(1), a5182. https://doi.org/10.4102/phcfm.v18i1.5182, for more information.

QARI, Qualitative Assessment and Review Instrument; PI, paediatric influence; SIP, Italian Paediatric Society.
