# Corrigendum: Evaluating the efficacy of digital media platforms in disseminating public health information: A global review with implications for South Africa

**DOI:** 10.4102/phcfm.v18i1.5425

**Published:** 2026-03-02

**Authors:** Hulisani Matakanye, Ndivhuwo D. Sundani

**Affiliations:** 1 Department of Health Studies, College of Human Science, University of South Africa, Pretoria, South Africa; 2 Department of Communication Science, College of Human Science, University of South Africa, Pretoria, South Africa

In the published article, Matakanye H, Sundani ND. Evaluating the efficacy of digital media platforms in disseminating public health information: A global review with implications for South Africa. Afr J Prm Health Care Fam Med. 2026;18(1), a5182. https://doi.org/10.4102/phcfm.v18i1.5182, there was an error regarding the affiliations for Hulisani Matakanye, and Ndivhuwo D. Sundani. The word *Faculty* should be *College*. Thus, the their full affiliation(s) should read:

Hulisani Matakanye:

Department of Health Studies, College of Human Science, University of South Africa, Pretoria, South Africa.

Ndivhuwo D. Sundani:

Department of Communication Science, College of Human Science, University of South Africa, Pretoria, South Africa.

The authors apologise for this error. The correction does not change the study’s findings of significance or overall interpretation of the study’s results or the scientific conclusions of the article in any way.

